# The clinical impact of molecular breast imaging in women with proven invasive breast cancer scheduled for breast-conserving surgery

**DOI:** 10.1007/s10549-018-4706-1

**Published:** 2018-02-13

**Authors:** Angela Collarino, Renato A. Valdés Olmos, Lotta G. A. J. van Berkel, Peter A. Neijenhuis, Lidy M. H. Wijers, Frederik Smit, Lioe-Fee de Geus-Oei, Lenka M. Pereira Arias-Bouda

**Affiliations:** 1 Section of Nuclear Medicine, Department of Radiology, Leiden University Medical Center, Albinusdreef 2, 2333 ZA Leiden, The Netherlands; 2 Biomedical Photonic Imaging Group, MIRA Institute, University of Twente, 217, 7500 AE Enschede, The Netherlands; 3 Interventional Molecular Imaging Laboratory, Department of Radiology, Leiden University Medical Center, Albinusdreef 2, 2333 ZA Leiden, The Netherlands; 4 Department of Nuclear Medicine, The Netherlands Cancer Institute–Antoni Van Leeuwenhoek Hospital, Plesmanlaan 121, 1066 CX Amsterdam, The Netherlands; 5 Department of Radiology, Alrijne Ziekenhuis, Simon Smitweg 1, 2353 GA Leiderdorp, The Netherlands; 6 Department of Surgery, Alrijne Ziekenhuis, Simon Smitweg 1, 2353 GA Leiderdorp, The Netherlands; 7 Department of Nuclear Medicine, Alrijne Ziekenhuis, Simon Smitweg 1, 2353 GA Leiderdorp, The Netherlands

**Keywords:** Breast cancer, Preoperative breast imaging, Molecular breast imaging, MBI, Breast-specific gamma imaging, BSGI

## Abstract

**Purpose:**

To investigate the clinical utility of molecular breast imaging (MBI) in patients with proven invasive breast cancer scheduled for breast-conserving surgery (BCS).

**Methods:**

Following approval by the institutional review board and written informed consent, records of patients with newly diagnosed breast cancer scheduled for BCS who had undergone MBI for local staging in the period from March 2012 till December 2014 were retrospectively reviewed.

**Results:**

A total of 287 women (aged 30–88 years) were evaluated. MBI showed T stage migration in 26 patients (9%), with frequent detection of in situ carcinoma around the tumor. Surgical management was adjusted in 14 of these patients (54%). In 17 of 287 patients (6%), MBI revealed 21 proven additional lesions in the ipsilateral, contralateral breast or both. In 18 of these additional foci (86%), detected in 15 patients, malignancy was found. Thirteen of these 15 patients had ipsilateral cancer and 2 patients bilateral malignancy. In total, MBI revealed a larger tumor extent, additional tumor foci or both in 40 patients (14%), leading to treatment adjustment in 25 patients (9%).

**Conclusion:**

MBI seems to be a useful imaging modality with a high predictive value in revealing ipsilateral and bilateral disease not visualized by mammography and ultrasound. It may play an important role in delineating the extent of the index lesion during preoperative planning. Incorporation of MBI in the clinical work-up as an adjunct modality to mammography and ultrasound may lead to better selection of patients who could benefit from BCS.

## Introduction

Invasive breast cancer is the most common cancer among women, with an incidence of 14.479 new cases in 2016 in The Netherlands [1] and 1.67 million new cancer cases diagnosed in 2012 worldwide [2].

In the last decades, breast-conserving surgery (BCS), also called lumpectomy, has gained importance due to the possibility to remove the tumor preserving the natural shape of the breast [3]. BCS is contraindicated in small breasts with large primary tumors and in case of multicentric tumors [3, 4]. Therefore, accurate definition of the extent of the primary tumor and exclusion of additional foci of cancer (multifocal, multicentric and contralateral breast cancer) is important in order to conduct the appropriate surgical treatment. Currently, magnetic resonance imaging (MRI) and molecular breast imaging (MBI) have been indicated to assess tumor extent and multifocal, multicentric and contralateral disease in adjunction to mammography (MG) and ultrasonography (US) [5, 6]. MBI is a functional imaging technique consisting of a breast-dedicated gamma camera equipped with a small field-of-view (FOV) single- or dual-head detector, producing high-resolution images corresponding to the standard projections used in MG [7–11]. In MBI, tumor-seeking radiopharmaceuticals like ^99m^Tc-sestamibi are used. Uptake of this tracer into tumor cells is based on increased vascularity and high mitochondrial density [12–14]. Recently, a low-dose protocol with an injected dose of 260–500 MBq ^99m^Tc-sestamibi has been introduced using a single-head MBI device [15, 16]. Dual-head MBI devices allow even lower injected doses varying from 150 to 300 MBq of ^99m^Tc-sestamibi [11, 17]. This leads to both reduction of absorbed dose and effective dose to the breast [18]. Compared to MRI, MBI is easy to interpret, is associated with low costs and is not contraindicated in patients with claustrophobia, overweight, implanted devices and renal insufficiency.

The purpose of this study was to investigate the clinical utility of MBI in adjunction to MG and US for delineation of the extent of the index lesion and to rule out additional tumor foci in patients with invasive breast cancer scheduled for BCS.

## Materials and methods

### Patients

The institutional review board approved this retrospective study and informed consent was obtained from all patients. Patients were included if they fulfilled the following criteria: (a) presence of histopathologically proven invasive breast cancer; (b) after conventional clinical work-up (including 2D MG, Siemens Inspiration Mammomat, and 2D US, Philips Affiniti 70 G Linear transducer L 12-5) the patient was scheduled for BCS; (c) the patient had undergone pretreatment MBI for assessment of tumor extent and presence of multifocal or multicentric disease; (d) complete individual data were available concerning clinical work-up, imaging, surgery and histopathology.

### MBI acquisition

MBI imaging was performed using a dedicated device equipped with a single detector system also known as breast-specific gamma imaging (BSGI; Dilon 6800, Dilon Diagnostics, Newport News, Virginia, U.S.A.). Images were acquired with the patient in seated position and with the breast in light compression. At our institution, we used a relative low-dose protocol (600 MBq) in comparison with the most published articles (740–1110 MBq) [7–9]. As mentioned earlier, recent studies showed that it seems possible to use even lower injected doses with BSGI [15, 16]. Each patient received an injection of approximately 600 MBq of ^99m^Tc-sestamibi into an antecubital vein contralateral to the breast lesion. Approximately 5–10 min after the injection, craniocaudal (CC) and mediolateral oblique (MLO) planar images were obtained for each breast, comparable with those of MG. The acquisition time for each image was 8–10 min, giving a total acquisition time of approximately 40 min per study. If relevant, additional planar images (lateromedial or mediolateral view, anteroposterior view (axilla) or axillary craniocaudal view) were acquired from the ipsilateral breast.

### MBI image analysis

All MBI images were evaluated by two nuclear medicine physicians of our institute (L.M.P.A-B and F.S.) and were directly compared with the most recent MG following the functional Breast Imaging Reporting and Data System (BI-RADS) classification [6, 19].

The size of the index lesion was calculated by measuring the maximum diameter (mm) of the pathological uptake on the MBI images (_MBI_ T stage). In case of more than one lesion, the maximum diameter of the largest tumor was used. Index lesion size detected on MBI was compared with the lesion size obtained with MG and US (_MORPHOLOGICAL_ T stage).

MBI-detected abnormalities were considered to be additional tumor lesions when they were suspicious on MBI (BI-RADS 4 or 5) and occult on MG and initially not picked up on US. At our institute, US is used to characterize a palpable mass or to find a correlate for a mammographical lesion. According to this criterion, the radiologist performed US of a sole lesion and not of a quadrant of the breast nor the whole breast. The additional breast lesions were classified as follows: (1) multifocal lesions when located in the same quadrant of the breast as the index tumor; (2) multicentric lesions when located in a different quadrant of the breast compared to the index tumor; and (3) contralateral lesions when located in the contralateral breast. The size of each additional lesion was measured on MBI corresponding to the maximum diameter (mm) of the pathological uptake. Histopathology was obtained from all additional MBI lesions after incisional needle-biopsy or surgical excision. The biopsy was performed using US-guided biopsy when the lesion was visible on targeted US. In more detail, after performing MBI and finding an additional suspicious lesion, the patient returned to the radiology department to undergo targeted US. In most cases, the additional detected lesion was previously not picked up during diagnostic work-up, since no routine whole breast screening US was performed. Targeted US was performed directly after MBI in case of an unexpected additional lesion, followed by US-guided biopsy. The radioactivity in the biopsy specimen was measured to prove that the lesion found on targeted US corresponded to the additional lesion found with MBI. In case of additional BI-RADS 4b,c or 5 lesions on MBI that remained occult on targeted US, MBI-guided biopsy was performed, but only if clinically relevant. BI-RADS 4a MBI abnormalities where considered benign if no correlate was found at targeted US.

### Statistical analysis

The *χ*^2^ test was used to analyze significant differences between dense and non-dense index lesion tumor as well as high-grade and low-grade. A *p* value of <  0.05 was considered statistically significant. Statistical analysis was performed using MedCalc Statistical Software version 15.11.4.

Based on T stage migration (upstaging) after MBI, the percentage of patients in who surgical management was adjusted based on the MBI results was calculated. Based on the biopsy-or excision-acquired pathological findings, all additional lesions with malignant histopathology like invasive tumor and ductal carcinoma in situ (DCIS) were considered true positive, while all additional lesions with benign histopathology were defined false positive. On the basis of the detected additional lesions on MBI, the lesion-based positive predictive value (PPV) was calculated using the formula True-positive/True-positive + False Positive  ×  100.

## Results

Records of 304 women with proven invasive breast cancer scheduled for BCS who underwent MBI between March 2012 and December 2014 were reviewed. Seventeen of these women, who had additional MBI-detected lesions without histopathological diagnosis, were excluded from the final analysis. In more detail, in 4 of these 17 patients the multidisciplinary team agreed upon that it was not necessary to prove the malignant nature of the lesion, because it was located nearby the index lesion and would not alter the treatment plan. Since these patients were treated with neoadjuvant therapy, it was not possible to verify the nature of the additional lesion afterwards. Thirteen of 17 patients had focal MBI lesions classified as BI-RADS 4a, meaning that there was doubt about the real nature of the MBI finding, for example because it was visible in only one view and could be caused by over-projection or an artifact. In these 13 patients, the finding was considered benign because no correlate was found with targeted US. The remaining 287 patients who fulfilled the inclusion criteria were enrolled in this retrospective study. The characteristics of the patients are reported in Table 1. The mean age of the patients was 60 years (range, 30–88 years). A significant difference was found between high-grade and low-grade breast tumors (*p* <  0.008), since more patients had low-grade tumors (grade 1 or 2). No significant difference was found between dense and non-dense breast tissue (*p *= 0.8) in this study population. The mean morphological maximum tumor diameter, obtained with MG and US, was 18 mm (range, 3–55 mm). In 246 patients (86%), the index lesion concerned invasive ductal carcinoma (IDC), in 24 patients (8%) invasive lobular carcinoma (ILC), and in 1 patient (0.3%) mixed IDC and ILC. The remaining 16 patients (5.7%) had other tumor types including 6 mucinous carcinomas, 3 papillary carcinomas, 3 apocrine carcinomas, 2 medullary carcinomas, and 2 tubular carcinomas.

**Table 1:** Title: Patient characteristics

Number of pts	287
Mean age (range)	60 (30–88)
Menopausal status	
Pre-/perimenopausal	79
Postmenopausal	208
Breast tissue composition*	
a	35
b	107
c	127
d	18
Mean tumor size (range)	18 mm (3–55 mm)
Multifocal/multicentric	18
T Stage prior to surgery	
T1a	6
T1b	63
T1c	128
T2	89
Unknown	1
Tumor type	
IDC	246
ILC	24
Mixed IDC/ILC	1
Other	16
Tumor subtype	
HER2-positive	40
ER-positive/HER2-negative	199
Triple negative	38
Scarff-Bloom Richardson Grade**	
1	43
2	112
3	124
Unknown	8

*No significant difference between dense (c, d) and non-dense (a, b) breast tissue (*p *= 0.8)

**Significant difference between high-grade (grade 3) and low-grade (grade 1, 2) breast tumors (*p* < 0.008)

^a^almost entirely fat; ^b^scattered fibroglandular density; ^c^heterogeneously dense; ^d^extremely dense; ^IDC^invasive ductal carcinoma; ^ILC^invasive lobular carcinoma; ^DCIS^ductal carcinoma in situ; ^ER^estrogen receptor; ^HER2^human epidermal growth factor receptor 2

**Fig. 1:**
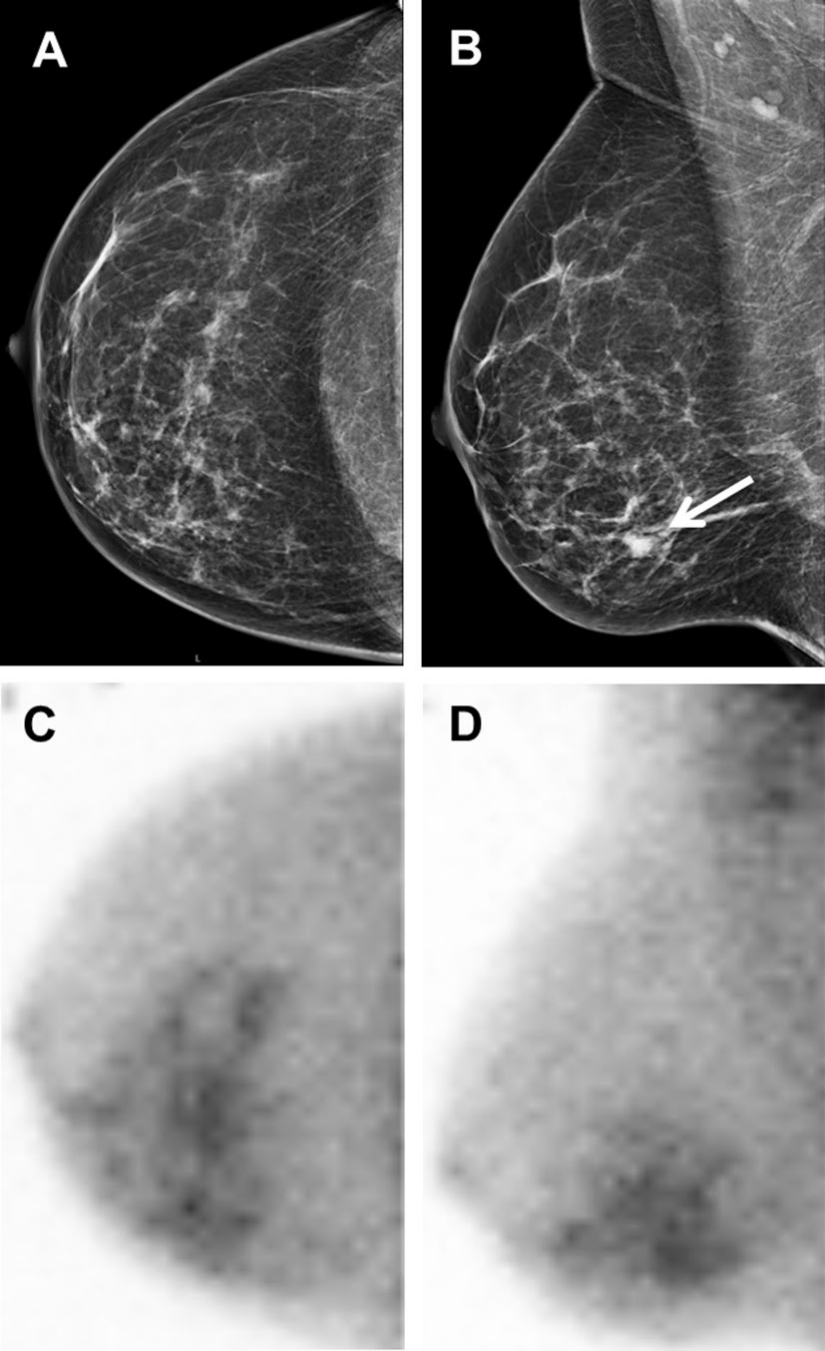
A 52-year-old woman (patient 13, Table 2) with invasive breast cancer. **a** Right craniocaudal mammographic image and **b** right mediolateral oblique mammographic image showing a breast mass of 10 mm with new calcifications in the lower inner quadrant of the right breast, best visible on the mediolateral oblique view (white arrow). **c** Right craniocaudal and **d** right mediolateral oblique MBI images showing a large and heterogenous area of pathological uptake (85 mm) in the lower inner quadrant of the right breast. The treatment changed from lumpectomy to mastectomy. Pathological findings revealed intracystic papillary adenocarcinoma and extralesional ductal carcinoma in situ with extension towards the nipple

Concerning the diameter of the index lesion, concordance between MBI and radiologic imaging was found in 261 patients (91%). In 26 out of 287 patients (9%), MBI showed T stage migration with adjustment of the surgical management in 14 of these 26 patients (54%) (Table 2). Five patients underwent unilateral mastectomy, 1 patient bilateral mastectomy, another 5 patients were treated with large lumpectomy, 2 patients received NAC before BCS, and 1 patient underwent quadrantectomy. In 10 of these 14 patients, the larger tumor extent on MBI was related to histopathologically proven DCIS around the invasive lesion (Fig. 1).

**Table 2:** T stage migration and treatment adjustment following MBI

N of pts	Morphological size (mm)	MBI size (mm)	T stage migration	Treatment plan before MBI	Treatment plan after MBI
1	20	24	T1 > T2	BCS	BCS
2	20	24	T1 > T2	BCS	BCS
3	16	35	T1 > T2	BCS	Large BCS
4	17	30	T1 > T2	BCS	BCS
5	11	35	T1 > T2	BCS	Large BCS
6	50	55	T2 > T3	NAC + BCS	NAC + BCS
7	40	63	T2 > T3	NAC + BCS	NAC + BCS
8	19	40	T1 > T2	BCS	Large BCS
9	20	27	T1 > T2	BCS	Mastectomy
10	19	30	T1 > T2	BCS	Large BCS
11	17	30	T1 > T2	BCS	Large BCS
12	20	42	T1 > T2	BCS	NAC + BCS
13	10	85	T1 > T3	BCS	Mastectomy
14	23	26	T1 > T2	Left BCS	Mastectomy
	13	80	T1 > T3	Right BCS	Mastectomy
15	15	21	T1 > T2	BCS	BCS
16	20	32	T1 > T2	BCS	BCS
17	20	24	T1 > T2	BCS	BCS
18	10	26	T1 > T2	BCS	BCS
19	7	30	T1 > T2	BCS	Mastectomy
20	20	25	T1 > T2	BCS	BCS
21	40	120	T2 > T3	BCS	NAC + BCS
22	40	90	T2 > T3	BCS	Quadrantectomy
23	40	90	T2 > T3	BCS	Mastectomy
24	19	23	T1 > T2	BCS	BCS
25	16	23	T1 > T2	BCS	BCS
26	16	90	T1 > T3	BCS	Mastectomy

*N* number, *Pts* patients, *MBI* molecular breast imaging, *BCS* breast-conserving surgery, *NAC* neoadjuvant chemotherapy

**Fig. 2:**
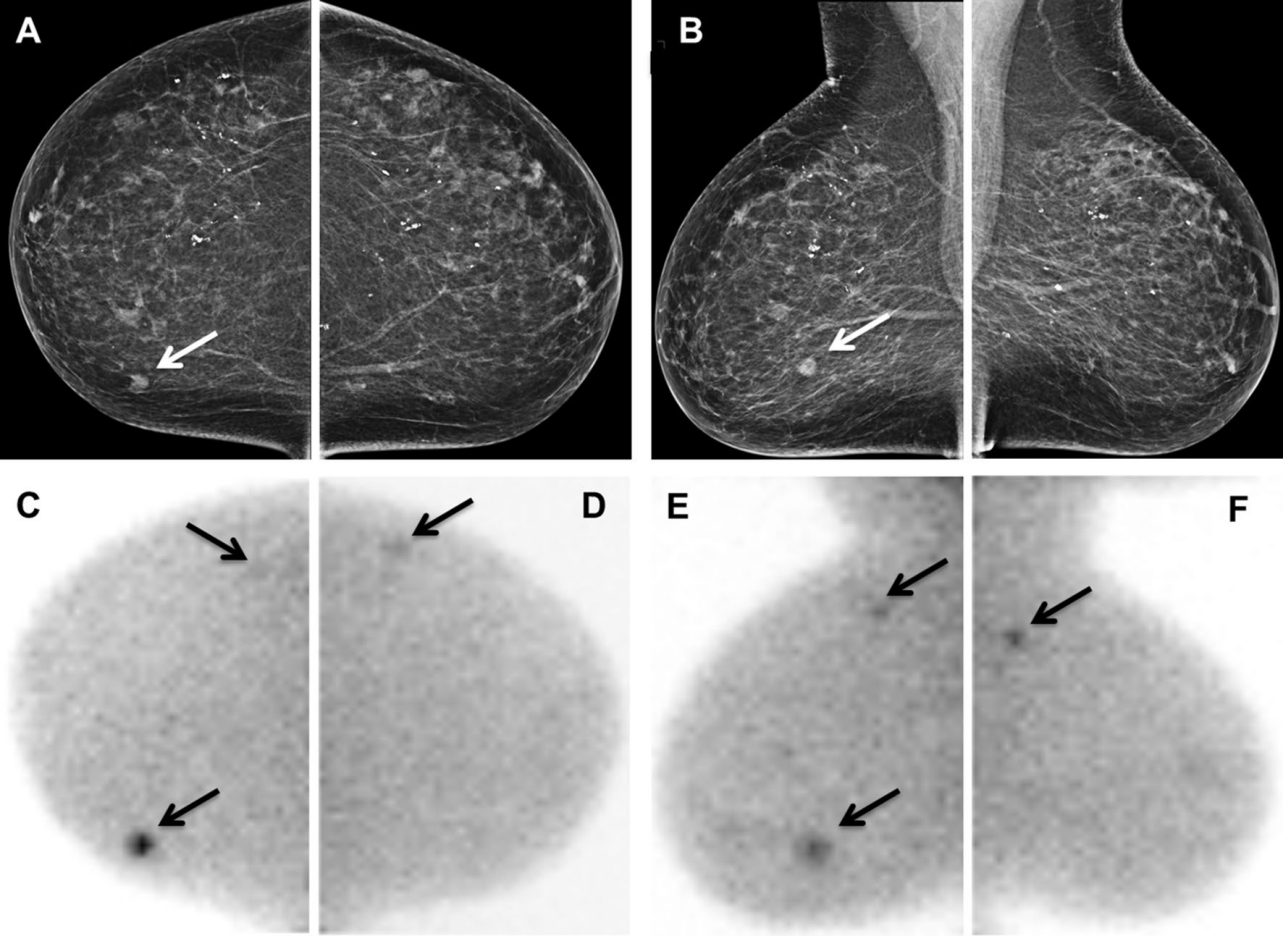
A 71-year-old woman (patient 1, Table 3) with invasive ductal carcinoma of the right breast and two additional tumor foci (1 in ipsilateral breast and 1 in contralateral breast). **a** Right craniocaudal and **b** right mediolateral oblique mammographic images showing a mass of 9 mm in the lower inner quadrant of the breast (white arrows). **c** Right craniocaudal MBI image showing two foci with pathological ^99m^Tc-sestamibi uptake (arrows), one intense accumulation medially corresponding to the mass seen on mammography and a mild accumulation laterally corresponding to the new 8-mm lesion located in the upper outer quadrant (multicentric lesion). **d** Left craniocaudal MBI image shows a new mild focal accumulation in the upper outer quadrant (arrow). **e** Right mediolateral oblique MBI image shows two intense foci (arrows): a caudal accumulation corresponding to the mass seen on mammography and a cranial accumulation (8 mm) corresponding to the new lesion located in the upper outer quadrant (multicentric lesion). **f** Left mediolateral oblique MBI image showing a focal intense accumulation of 7 mm in the upper outer quadrant (contralateral lesion). For both additional lesions, the patient underwent US-guided biopsy after targeted US that revealed invasive ductal carcinoma in the additional lesion in the right breast and ductal carcinoma in situ in the additional lesion in the left breast. The treatment changed from local excision (right breast) to bilateral mastectomy

In 17 of 287 patients (6%), MBI revealed 21 proven additional lesions in the ipsilateral breast, contralateral breast or both breasts (Table 3). The median size of these lesions on MBI was 10 mm (range: 7–35 mm). Histopathological features were obtained by needle-biopsy from 16 out of 21 lesions in 12 patients and by surgical excision from the other 5 lesions concerning another 5 patients. Breast needle-biopsies were performed under guidance of US in 10/12 patients and under MBI guidance in 2/12 patients. In 15 out of these 17 patients (88%) the 18 additional lesions turned out to be malignant. In 13 of these 15 patients, the malignant lesions concerned ipsilateral tumors (15/18 lesions) and in 2 patients bilateral tumors (3/18 lesions) (Fig. 2). The surgical management was adjusted in 12 out of these 15 patients (80%). In more detail, 3 patients were converted to NAC and mastectomy, while 7 patients underwent ipsilateral mastectomy instead of BCS, 1 patient was treated with bilateral mastectomy, and 1 patient with bilateral BCS. From the 18 additional proven malignant lesions (true positives) on MBI, 6 lesions were smaller than 10 mm (range: 6-8 mm). The pathologic findings of the 18 tumors included 10 IDC, 3 ILC, and 5 DCIS. The remaining 3 additionally detected lesions on MBI were benign lesions (false positives) revealing mastopathy in one, one with fibroadenoma, and one with a mixed pattern of mastopathy and adenosis. The MBI lesion-related PPV was 86%.

**Table 3:** Additional suspicious lesions detected on MBI, occult on MG and US

N of pts	N of additional lesions	Size (mm)	Multifocal lesions	Multicentric lesions	Contralateral lesions	Biopsy/surgical excision	Malignant lesions	Benign lesions	Treatment plan before MBI	Treatment plan after MBI
1	2	8 R;7 L		1 R	1 L	US-guided	IDC and DCIS		BCS	Bilateral mastectomy
2	1	35		1		MBI-guided		Mastopathy and adenosis	BCS	BCS
3	1	8	1			US-guided	IDC		BCS	BCS
4	1	7	1			US-guided		Mastopathy	BCS	BCS
5	2	12; 11		2		US-guided	2 ILC		NAC and BCS	NAC and mastectomy
6	1	8	1			Excision	DCIS		BCS	BCS
7	1	7	1			Excision	DCIS		BCS	BCS
8	1	10		1		Excision	DCIS		BCS	Mastectomy
9	1	11		1		US-guided	IDC		BCS	Mastectomy
10	1	6		1		Excision	DCIS		BCS	Mastectomy
11	1	11		1		US-guided	IDC		BCS	Mastectomy
12	1	26	1			MBI-guided	IDC		NAC and BCS	NAC and mastectomy
13	1	14	1			Excision	IDC		BCS or mastectomy	Mastectomy
14	2	10; 30	1	1		US-guided	2 IDC		NAC and BCS	NAC and mastectomy
15	2	12; 12			2 L	US-guided	IDC	Fibroadenoma	BCS	Bilateral BCS
16	1	10		1		US-guided	IDC		BCS	Mastectomy
17	1	10	1			US-guided	ILC		BCS	Mastectomy

*MBI* molecular breast imaging, *R* right breast, *L* left breast, *IDC* invasive ductal carcinoma, *ILC* invasive lobular carcinoma; *DCIS* ductal carcinoma in situ; *BCS* breast conservative surgery, *NAC* neoadjuvant chemotherapy

**Fig. 3:**
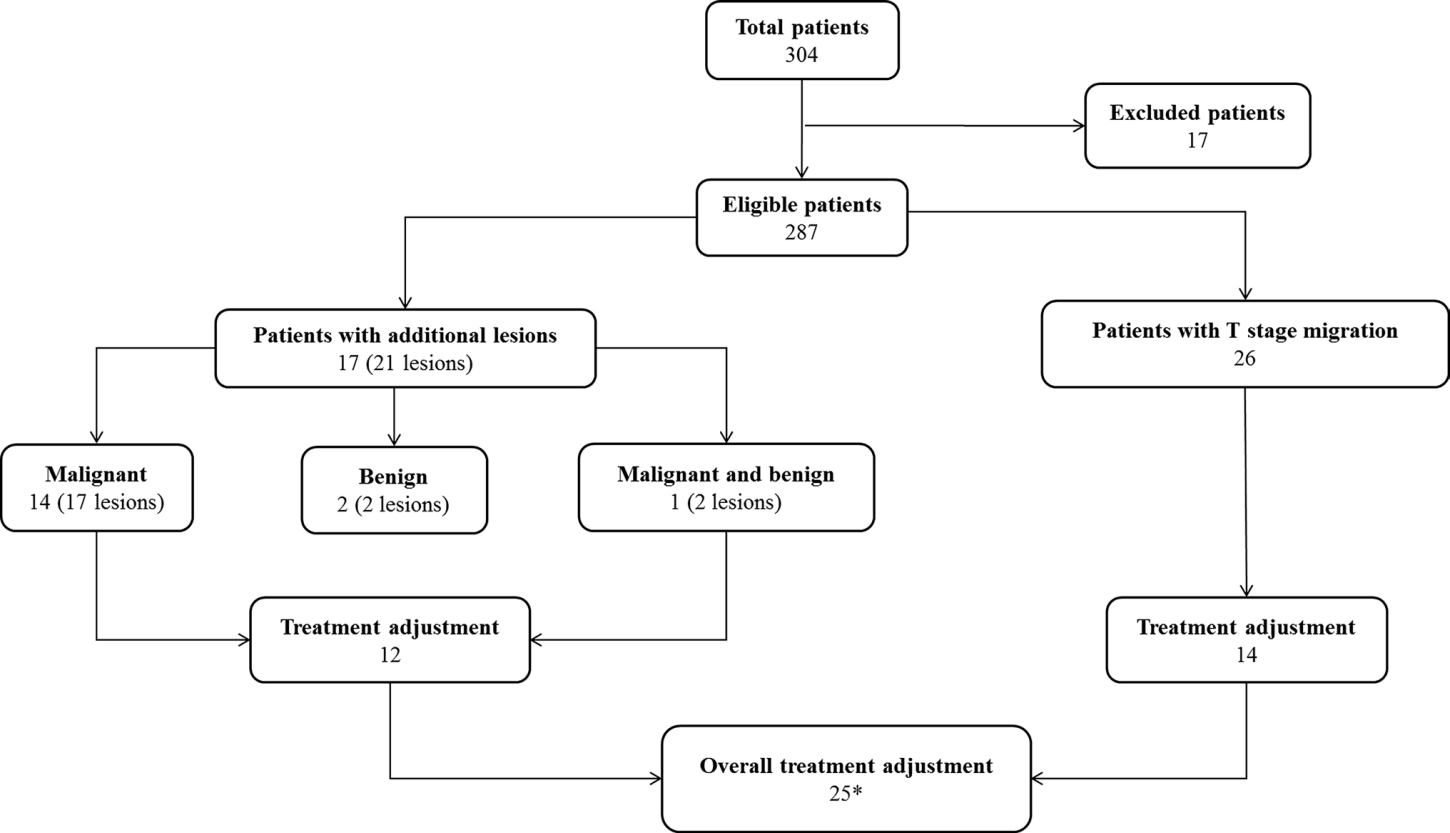
Flowchart showing the impact of preoperative MBI in the study population. *One patient had both T stage migration as well as an additional malignant lesion on MBI

Overall, MBI showed an unexpected larger tumor extent, additional tumor foci or both in 40 of 287 included patients (14%). In one of these 40 patients, MBI revealed both a larger index tumor as well as multicentricity (patient nº19 in Table 2 and n°10 in Table 3). In 4 of these 40 patients (10%), the index lesion concerned ILC. Twenty patients (50%) had non-dense breast tissue with breast composition a in 4 and breast composition b in 16 patients. Owing to the use of MBI, the overall treatment was adjusted in 25 patients (9%) (Fig. 3).

## Discussion

In the present study, we evaluated the clinical impact of ^99m^Tc-sestamibi MBI, incorporated in the diagnostic work-up of patients with newly diagnosed breast cancer scheduled for BCS, as an adjunct modality to MG and US. Based on our data, the preoperative use of MBI in this specific patient population resulted in the detection of a significantly larger disease extent, additional tumor foci or both in 14% of patients, albeit 50% of these patients had non-dense breast tissue on MG and only 10% had lobular type of carcinoma. The unexpected MBI findings led to treatment adjustment in 9% of all patients.

Evaluation of the extent of the index lesion using MBI showed a change of the local stage in 26 patients (9%) and local excision was abandoned in 14 of these patients. In our series, a larger disease extent as detected on MBI was mainly due to the visualization of DCIS located around the invasive tumor, which is in concordance with the findings of Spanu et al. [20]. Interestingly, in the majority of these patients no calcifications were found in the DCIS area on MG. Although it is not possible to distinguish carcinoma in situ from invasive tumor based on the ^99m^Tc-sestamibi uptake pattern, the total area of pathological ^99m^Tc-sestamibi uptake guided our surgeons during the surgical procedure, increasing the rate of complete surgical treatment and avoiding additional surgeries. Therefore, we postulate that MBI offers the possibility to plan resection of the index lesion more accurately based on the extension of ^99m^Tc-sestamibi uptake.

Additional lesions were visualized on MBI in 17 women (6%). Two of these patients underwent MBI-guided biopsy since the additional lesions remained occult even after targeted US. MBI-guided biopsy is a biopsy modality approved by the U.S. Food and Drug Administration (FDA) in 2009. This tool is based on stereotactic localization of the ^99m^Tc-sestamibi avid lesion [21, 22] and is currently used in our clinical work-up [23]. In our series, MBI-detected lesions corresponded to additional proven tumors in 15 women (5% overall detection rate). This is in line with the results reported in previous studies in the literature [20, 24–29]. Lesion-related analysis demonstrated that 18 out of 21 additional lesions visualized on MBI resulted in true cancer. The high PPV of 86% suggests that a positive MBI scan is highly predictive for occult tumor. This is in concordance with the relative high specificity of this technique as described in the literature [30]. A possible explanation is the highly specific uptake of ^99m^Tc-sestamibi by tumor cells as compared to the surrounding breast tissue [12–14]. Moreover, MBI detected 6 subcentimeter additional cancers in our series. This agrees with prior studies [20, 27, 28] reporting the ability of MBI to identify occult tumors smaller than 1 cm. Recently, new MBI systems based on dual-head cadmium-zinc-telluride (CZT) detectors have been introduced offering improved sensitivity for detection of small tumors [11]. In our series the detection of additional tumors on MBI has led to the abandonment of lumpectomy in 11 women. Combining the contribution of MBI in relation to investigating disease extent and presence of multifocal and multicentric disease, the overall management was adjusted in 9% of all patients with newly diagnosed BC scheduled for lumpectomy. Additionally, MBI showed a low false-positive rate, thus avoiding unnecessary biopsies, complementary imaging, and patient anxiety.

In the light of our results, MBI could be a valid adjunct modality to MG and US for detecting both the extent of index lesions and additional tumor foci. Presently, MRI is widely used in the clinical work-up of newly diagnosed breast cancer in women. Although MRI shows high sensitivity, its low specificity and high costs limit a wide application of this modality. Additionally, MRI is not applicable in patients with claustrophobia, overweight, implanted devices and renal insufficiency [31]. MBI has the potential to overcome these limitations becoming a useful tool for almost all newly diagnosed breast cancer patients. Additionally, MBI is easy to perform and is associated with low costs. On the other hand, MBI requires the intravenous injection of a radiotracer, like ^99m^Tc-sestamibi, which means radiation exposure for the patient. However, one should keep in mind that the administered dose of ^99m^Tc-labeled sestamibi for MBI is similar to the dose used for other commonly applied diagnostic functional imaging examinations such as bone scintigraphy and myocardial perfusion imaging [32, 33]. Moreover, recent technological advances in MBI allow a significant reduction of the injected dose of the radiopharmaceutical. Indeed, it is possible to use a low-dose imaging protocol with CZT-based dual-head MBI (150–300 MBq) [11, 17] as well as with NaI-based single-head MBI (260–500 MBq) [15, 16]. An administered dose of 150 MBq ^99m^Tc-sestamibi leads to a significant reduction of absorbed dose to the breast (0.25 mGy) and effective dose (1.1 mSv) [18].

Finally, it is necessary to address the principal limitations of the present study. First, it concerns a retrospective study based on data collected in a single institution. Second, we retrospectively excluded a relative large amount of patients with positive MBI studies due to missing histopathological data. This represents a potential bias of the presented MBI results. However, the excluded cases represented either patients in who the unexpected detection of additional lesions was not clinically relevant (in the sense that it would not have altered the treatment plan), or patients with a relatively low probability of having additional malignant foci (BI-RADS 4a abnormalities without correlate at targeted US). Third, an injection dose of 600 MBq was applied using a single-head detector. Since others have found comparable results using low-dose protocols (260–500 MBq for single-head MBI or 150–300 MBq for dual-head MBI) versus high-dose protocols [15–17], it would be worthwhile to investigate the performance of the low-dose protocol in the studied patient population, since it could lead to a significantly lower radiation exposure. On the other hand, the strength of this study is that it represents the first series evaluating the additional clinical value of MBI in a large population of patients with proven invasive breast cancer scheduled for breast-conserving surgery.

## Conclusion

The results of the present study support that MBI is a useful imaging modality, which reveals a high rate of multifocal or multicentric lesions and bilateral disease not visualized by mammography and ultrasound. Additionally, MBI may play an important role in accurate delineation of the tumor extent during preoperative planning. Therefore, the incorporation of this modality to the clinical work-up may lead to better selection of patients who might benefit of BCS. However, larger and prospective studies, preferably using low-dose MBI protocols, are needed to confirm these findings.
